# Partial Suppurating Pulpitis—Origin of Certain Alveolar Abscesses in Living Teeth

**Published:** 1892-10

**Authors:** Arthur C. Hugenschmidt

**Affiliations:** Paris, France


### PARTIAL SUPPURATING PULPITIS—ORIGIN OF CERTAIN ALVEOLAR ABSCESSES IN LIVING TEETH.[1]

BY ARTHUR C. HUGENSCHMIDT, M.D., D.D.S., PARIS, FRANCE.

CHRONIC localized suppurating pulpitis, that is, a *suppurating* inflammatory process restricted to a portion only of the pulp, such as we commonly observe in teeth in which the pulp has been exposed for a long time, protected only by alimentary particles packed in a carious cavity, can also exist in a tooth in which no communication has ever existed, artificially by instruments or otherwise by natural pathological processes, between the external surface of the tooth or crown and the pulp-cavity. I refer, it must be well understood, to a *localized suppurating* inflammation, and to no other localized non-suppurating inflammatory process which is found in living pulps. In such non-penetrating carious teeth, where such a pulp-lesion exists, provided the conditions I shall hereafter describe are present, the pathological state will manifest itself under the form of a chronic alveolar abscess opposite the end of a single rooted tooth while the pulp of that same tooth is still alive, as it answers to all the principal physiological reactions.

In adult teeth we find such conditions rarely, yet I am convinced that every careful practitioner has certainly met with teeth presenting a fistulous opening opposite the apex of a certain tooth which the operator considered as dead; yet when he attempted to reach the pulp-cavity, to his amazement he finds a highly-developed sensitive dentine; if the neighboring teeth are tested in the same way, they also are found alive, and yet the first tooth is the real seat of the abscess.

While this condition is a rare one in the adult, *per contra* we find it much more frequent in the child, which is explainable, anatomically, by the fact that the apical foramen is very large, as a result of physiological radicular absorption, and I think that a large foramen in the adult, as well as in the child, is a necessity to enable the inflammatory products to make their exit from the pulp-cavity without injuring or destroying the vitality of the dental pulp.

The clinical facts which I have observed on several patients have been as follows:

The patient will present himself suffering from a chronic alveolar

---

[1] Read before the New York Odontological Society, June 21, 1892.

abscess, an ordinary gum-boil, which might have existed for months, and which is situated at a point on the gum opposite the apex of the root or roots of a tooth. The patient will complain that this abscess opens every day, or every two days, that the rupture of the same is preceded by a short period during which the tooth is sore, is annoying, a pressure on the same relieving the annoyance, but as soon as the abscess opens, everything, all physical symptoms disappear.

On clinical examination of the mouth we find a small, typical, alveolar abscess, about the size of a small pea, rolling under the finger, and situated opposite the apex of the root of a tooth. Examining the corresponding tooth, we find it normal, no caries being present, perhaps a small gold filling. On careful examination, however, the tooth opposite which the abscess exists may be slightly darker in color than the neighboring teeth, but very little so. Of course, the first step in the treatment of such a case, after having excluded in our diagnosis the alveolar abscess of external origin, due to an infectious alveolar arthritis, will be to reach and open the pulp-cavity in which we expect to find a dead pulp; but as soon as the drill has passed the limits of the enamel and penetrated the dentine the patient will begin to complain, and the more we advance the greater the pain becomes, so that, at a certain moment, the pain being unbearable, we feel convinced of having made a false diagnosis, and the cavity artificially formed will be closed with gutta-percha or some other material.

Turning our attention to the neighboring teeth, and, *imagination aiding, we think* that the second one nearest to the fistula is also a little darker, so on that tooth the instrument goes, and as soon as the enamel is passed, we find again sensitive dentine; if the tooth on the other side be opened, also, the same positive results as regards sensibility will be noted.

Now we find ourselves in presence of three sensitive teeth, sensitive not only to the touch, to instruments, but also to extremes of temperature, heat and cold; teeth which respond to a normal sensibility, to a normal state, and yet one of those organs, somewhere, is the cause of this persistent abscess.

Well, if, following our first impulsion—and a first impulsion is usually the good one—we continue our drilling in the first tooth which attracted our attention until we reach the pulp, we shall fall, as may be expected, on a living pulp, and a lively one, too; and if we are careful we shall find in the blood, escaping as a result of the instrumental lesion of the pulp-tissues, a small quantity of pus, which certainly comes from the pulp-cavity, and from the pulp in particular. It is in such cases that the pulp can be seen beating synchronously with the heart's action, as in such cases it is larger than usual, and the foramen apicale being also very large, *a fortiori*, the blood-vessels of the pulp will be more developed, and any other normal physiological act pertaining to the pulp will be magnified.

If, now, this pulp be removed without tearing, which can be done by injecting cocaine in the gums beforehand, so that the patient be very quiet, we shall be able to observe an ulceration of variable size,—usually a small pin's head,—situated somewhere on the surface of the pulp-organ, usually near the crown, but have also seen it on the radicular portion. Moreover, and this is an important fact, the apical foramen will be found unusually large, and for this reason, as well as for reasons given above, the bleeding produced by removal of the pulp is a profuse one, as if a comparatively large artery had been opened; it can be arrested, however, by tamponing the pulp-cavity.

It will be readily understood that this ulcerative process giving rise to suppuration, the pus will travel all along the external portion of the pulp until it reaches the apical foramen, which, being very large, will prevent strangulation of this organ, which otherwise would occur; from the apical foramen to the outside of the gum the pus will follow exactly the same course as it would in an ordinary alveolar abscess.

In the child, as I indicated above, the phenomenon of radicular absorption increasing much beyond normal the dimension of the foramen apicale, there exists a free communication between the pulp and the peridental tissues; and any localized pathological lesion of the pulp, if it passes at the period of suppuration, will allow its inflammatory products to pass with greater facility in the surrounding tissues through the apex than through the hard tissues of the tooth, which cannot yield unless there should be a penetrating carious cavity.

In the child, clinically, we observe an alveolar abscess larger than in the adult, attaining usually the size of a hazel-nut, and having for its origin a carious temporary tooth, the caries having not exposed the pulp-cavity. If we take away any dentine, the little patient will protest; also, if we inject cold or hot water, which is an indication of the existence of a living tooth; in addition, if we continue our operation, and open the pulp-cavity, we shall find a normally sensitive organ, which will bleed, and at the same time pus will be seen mixed with the blood, which is indicative of a neighboring suppurating lesion.

Lesions of the pulp have been indicated and carefully studied in a patho-histological point of view by Dr. Arnim Rothmann, of Buda-Pesth, who divided pulp-lesions into: 1. Acute partial pulpitis. 2. Acute purulent partial pulpitis. 3. Acute total pulpitis. 4. Total suppurating chronic pulpitis. According to that author, the first one is due to an inflammatory process which commences under the odontoblastic layer, and only extends to a very limited region. This inflammation is characterized by a cellular infiltration, which is probably due to micro-organisms. The second form is an ulcerous process, which also destroys the odontoblastic layers, and which, according to the duration of the disease, extends more or less deeply into the tissues of the pulp; if this second form lasts too long it becomes a total acute pulpitis. Finally, total suppurating chronic pulpitis is a chronic process, beginning in the centre of the pulp and extending slowly to the periphery. The diagnosis of these different lesions have been verified, according to the author's statement, by extraction of the involved teeth.

Dr. Rothmann does not mention the existence of a *chronic localized* suppurating pulpitis, and yet I am convinced that such is the lesion observed whenever a pulp has been long exposed and has not died, and also that such was the lesion which I observed in living teeth presenting abscesses, the histories of some of which I sent to the American Dental Association in August, 1890.

This chronic localized suppuration does not offer very characteristic clinical signs, and to establish our diagnosis we must satisfy ourselves with the three following facts: First, the presence of a chronic alveolar abscess, or dental fistula, opposite the apex of the root of the suspected tooth; secondly, to a more or less deep discoloration of the crown of the tooth as compared to the neighboring ones; thirdly, the indication given by the patient that the suspected tooth is the seat of an annoyance, of an obscure pain, when the abscess was forming.

When these three signs are present, and the neighboring teeth are not devitalized, one must not hesitate to trephine the suspected tooth, notwithstanding the dentinal sensibility which will be encountered, and surely, if the pulp be carefully removed, the origin of the trouble will be found.

As a conclusion, I think I can state that living teeth in adults, as well as in children, can give rise to an alveolar abscess.

